# Ovarian endometrioma: a report of a pediatric case diagnosed prior to menstruation

**DOI:** 10.1186/s40792-024-01951-5

**Published:** 2024-06-20

**Authors:** Lynne Takada, Takafumi Kawano, Keisuke Yano, Yumiko Iwamoto, Masato Ogata, Chihiro Kedoin, Masakazu Murakami, Koshiro Sugita, Shun Onishi, Mitsuru Muto, Mari Kirishima, Akihide Tanimoto, Satoshi Ieiri

**Affiliations:** 1 Department of Paediatric Surgery, Research Field in Medicine and Health Sciences, Medical and Dental Sciences Area, Research and Education Assembly, Kagoshima University, 8-35-1, Sakuragaoka, Kagoshima City, 890-8520 Japan; 2 Department of Pathology, Research Field in Medicine and Health Sciences, Medical and Dental Sciences Area, Research and Education Assembly, Kagoshima University, Kagoshima, Japan

**Keywords:** Ovarian endometrioma (OE), Premenarcheal endometriosis, Pediatric endometrioma

## Abstract

**Background:**

Ovarian endometriomas (OEs) are rarely found in the pediatric population, especially before menstruation. We report a 6-year-old girl who was postoperatively diagnosed with OE before menstruation.

**Case presentation:**

A 6-year-old girl presented to a local pediatrician with abdominal pain and vomiting. Abdominal ultrasonography revealed a multilocular cystic lesion to the left of the bladder. Magnetic resonance imaging (MRI) revealed similar findings, with the contents of the cyst showing a low signal on T1-weighted imaging and a high signal on T2-weighted imaging. The patient was referred to our institution for further examination. Enhanced computed tomography (CT) showed a multilocular cystic lesion sized 56 × 44 × 30 mm with partial calcification. The left ovarian vein was dilated, suggesting the origin of the tumor to be the left ovary. Extirpation of the lesion was performed under laparoscopic assistance. Pathological findings indicated an ovarian endometrioma. To our knowledge, this is the youngest report of an OE diagnosed in a patient prior to menstruation.

**Conclusions:**

OEs in children before menstruation are extremely rare; thus, the long-term prognosis is yet to be determined.

## Background

Endometriosis, a complex condition characterized by the ectopic presence of endometrial glands and stroma outside the confines of the uterine cavity, manifests as multifocal lesions in a myriad of pelvic and extrapelvic anatomical sites [1, 2]. Lesions are found in the ovaries, pelvic peritoneum, and various pelvic organs, including the bladder, ureter, rectum, sigmoid colon, appendix, and the fallopian tube. They can also be found outside the pelvis, such as in the vulva, umbilicus, lungs, pleura, kidneys, or at surgical sites.

Endometriosis affects approximately 10% of women of reproductive age [2]. Nevertheless, its actual prevalence is likely underestimated, owing to the exigency of definitive diagnosis through either laparoscopic visualization or histopathological scrutiny [3]. Previous studies report an estimated 6 of 10 endometriosis cases to be undiagnosed [4, 5]. Another study reports the prevalence of up to 22% among asymptomatic patients [1, 6]. The prevalence in children remains unknown, with only nine cases reported specifically in children before menstruation.

We herein report a rare case of a 6-year-old girl who was postoperatively diagnosed with an Ovarian Endometrioma (OE). To the best of our knowledge, this is the youngest report of OE diagnosed prior to menstruation.

## Case presentation

**Fig. 1:**
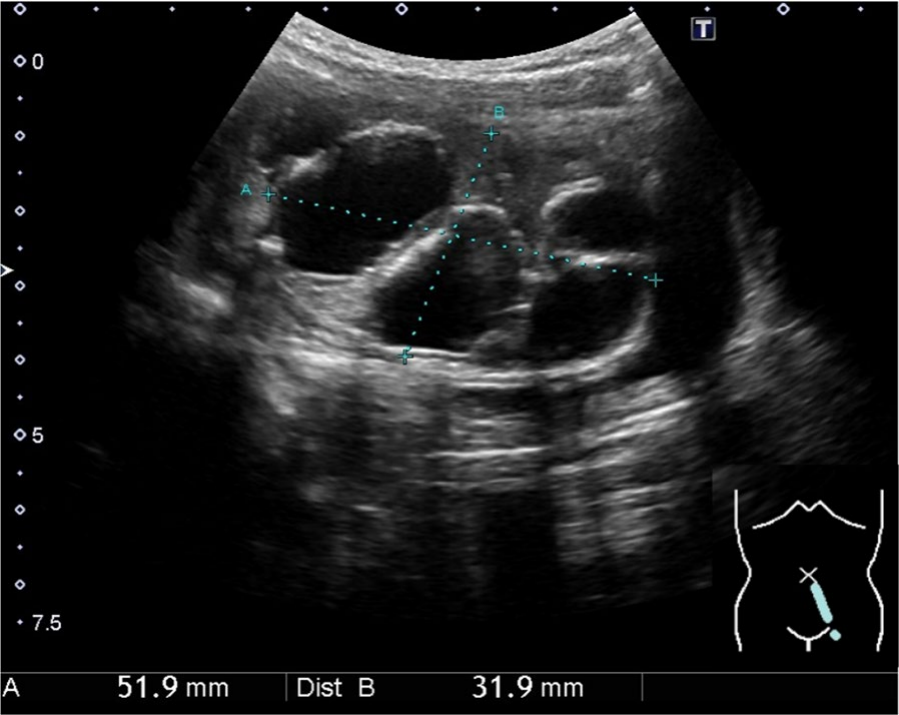
Abdominal ultrasonography findings. Abdominal ultrasonography revealed a multilocular cystic lesion to the left of the bladder, measuring 50 × 30 mm

**Fig. 2:**
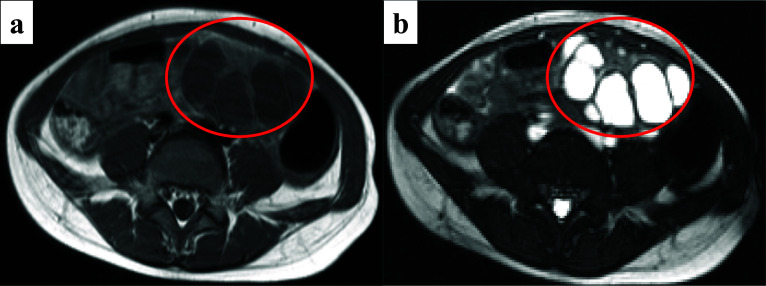
MRI findings. **a** Contents of the cyst showed low signal intensity on T1-weighted imaging. **b** Contents of the cyst showed high signal intensity on T2-weighted imaging

A 6-year-old girl presented to a local pediatrician complaining of abdominal pain and vomiting that had persisted since the previous night. She was diagnosed with acute infectious gastroenteritis, and her symptoms ceased with conservative treatment. During abdominal ultrasonography, a multilocular cystic lesion adjacent to the left side of the bladder (Fig. 1) was incidentally found. Magnetic resonance imaging (MRI) corroborated these findings, demonstrating low signal intensity on T1-weighted imaging (Fig. 2a) and high signal intensity on T2-weighted imaging (Fig. 2b) within the cystic contents. The patient was subsequently referred to our institution for further evaluation regarding the cystic lesion.

On examination, vital signs were within normal limits, the abdomen soft with no palpable mass. General blood test results were normal. However, tumor marker levels were as follows: cancer antigen 125 (CA125) 108.6 U/mL (reference range 0 to 30 U/mL), neuron-specific enolase (NSE) 19.1 ng/mL (reference range up to 16 ng/mL), α-fetoprotein (AFP) 1.4 ng/mL (reference range up to 10 ng/mL), and β-human chorionic gonadotropin (β-HCG) ≦0.1 ng/mL.

**Fig. 3:**
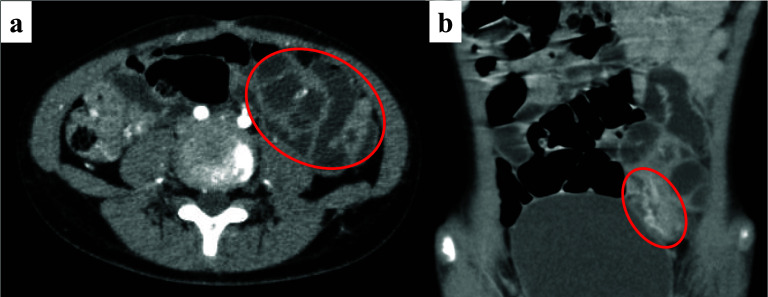
Enhanced CT findings. **a** Multilocular cystic lesion of 56 × 44 × 30 mm in size with partial calcification. **b** Dilated left ovarian vein

Enhanced computed tomography (CT) revealed a multilocular cystic lesion measuring 56 × 44 × 30 mm with partial calcification (Fig. 3a). In addition, dilation of the left ovarian vein was noted (Fig. 3b), suggesting the origin of the lesion to be the left ovary. Based on these imaging findings, a teratoma was considered the most probable differential diagnosis.

**Fig. 4:**
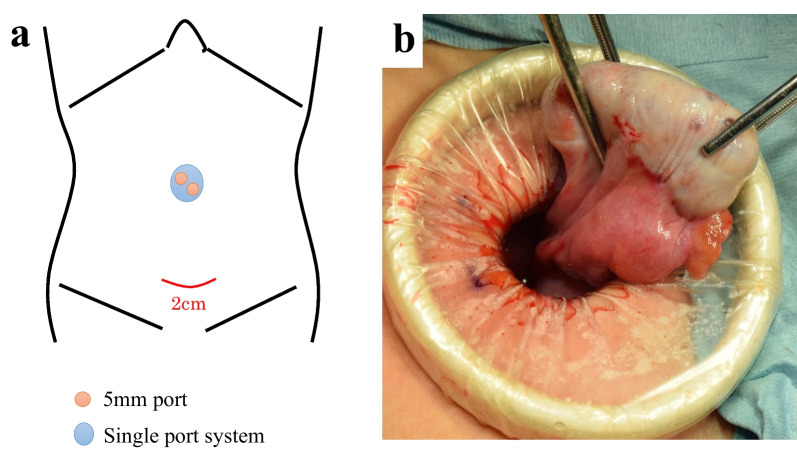
Surgical findings. **a** Port layout; two 5 mm ports in the umbilicus, a 20 mm length Pfannenstiel’s incision in the lower abdomen. **b** Intraoperative findings: Macroscopically, the white solid mass contained slightly tan-coloured serous fluid

Therefore, we planned a laparoscopy-assisted ovarian tumor extirpation. The port layout and intraoperative findings are shown in Fig. 4. Laparoscopic exploration of the abdominal cavity revealed no peritoneal lesions apart from the ovary itself. A small skin incision was made in the lower abdomen to facilitate observation and extirpation of the ovarian tumor. After careful extraction from the incision site, tumor extirpation was performed. Macroscopically, the white solid mass contained slightly tan-colored serous liquid. The postoperative course was uneventful, and the patient was discharged on postoperative day 5. CA 125 levels normalised a month after surgery.

**Fig. 5:**
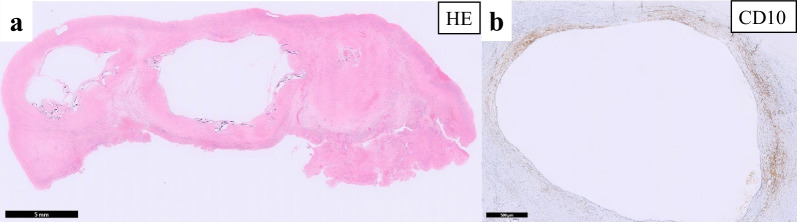
Histopathological findings. **a** Microscopically, the cyst was multilocular and calcification was seen along the cyst wall. **b **Immunohistochemistry showed CD10-positive spindle cells lining the walls of the cyst

Microscopically, the cyst was multilocular and calcification was seen along the cyst wall (Fig. 5a). The inner surface of the cyst wall was focally covered by cuboidal epithelium, but the epithelial lining was inconspicuous in most parts of the wall. Aggregation of hemosiderin-laden macrophages and foreign body-type multinucleated giant cells was also seen. Immunohistochemistry detected CD10-positive spindle cells lining the walls of the cyst (Fig. 5b). Based on these pathological findings, the lesion was diagnosed as an OE.

It has been 5 years since the surgery, and there have been no recurrences or symptoms indicative of endometriosis.

## Discussion

Endometriosis affects approximately 10% of women of reproductive age [2]. OEs are found in 17–44% of all adult patients with endometriosis [7, 8], comprising 35% of all benign ovarian cysts [7, 8]. Meanwhile, the prevalence of OEs in children remains uncertain, as most ovarian cysts in pediatric patients are serous or mucinous cystadenomas or mature teratomas. It is especially rare for a child to develop an OE prior to menstruation. There were nine cases of endometriosis diagnosed before menstruation reported in literature, aged between 8 and 13 years, as shown in Table 1. In 1996, Reese et al. reported cases of endometriosis before menarche in two adolescents aged 12 and 13 [9, 10]. Marsh et al. reported a series of five premenarcheal girls with pelvic pain who were found to have visual lesions consistent with endometriosis on laparoscopic evaluation [3]. Two cases in this study were reported of recurrence after menarche, through a second laparoscopic procedure performed for worsening pelvic pain unresponsive to hormonal therapy received since the onset of menarche. Reports of OE before menstruation are limited, with only two cases noted. Gogacz et al. described an 11-year-old adolescent postoperatively diagnosed with left OE [1]. Uchida et al. reported a case of OE in a 12-year-old girl before menarche [11]. To our knowledge, this is the youngest report of an OE diagnosed in a patient prior to menstruation.

**Table 1:** Review of 9 cases of premenstrual endometriosis reported in the literature

Author	Years	No. of cases	Age	Location	Symptoms	Modality	Treatment	Recurrence
Reese et al. [9]	1996	2	12	Peritoneum	Chronic Pelvic pein	Laparoscopy	N.A	N.A.
			13	Peritoneum	Chronic Pelvic pein	Laparoscopy	N.A	N.A.
Marsh et al. [3]	2005	5	8.5–13	Peritoneum	Chronic pelvic pain	Laparoscopy	Resection, Laser, OCPs	2 cases
Gogacz et al. [1]	2012	1	11	Ovary	Vomiting	Laparoscopy	Cystectomy only	None
Uchida et al. [11]	2017	1	12	Ovary	Abdominal pain	Laparoscopy	Cystectomy only	N.A.

*OCPs* low-dose oral contraceptive pills, *N.A.* not available

It is difficult to initially include an OE in the differential diagnosis of pediatric ovarian cysts, particularly before the onset of menstruation. All prior cases were postoperatively diagnosed through exploratory laparoscopy intended for diagnostic and therapeutic purposes. In individuals with OEs, including pediatric patients, surgical intervention alone is deemed insufficient owing to the potential persistence of microscopic residual disease, necessitating adjunctive medical suppression [6, 12]. In addition, evidence suggests that endometriosis may gradually progress in adolescents who do not adhere to medical therapy [1, 13]. Younger age has been reported as an independent risk factor for endometriosis recurrence after conservative surgical treatment of endometriosis in adults. [12, 14]. However, there are no reports regarding the long-term prognosis or preferred post-operative treatment of OEs diagnosed before menstruation.

Therapeutic intervention not only provides relief from clinical symptoms but also prevents the progression of endometriosis and future infertility. The first-line medical therapy for adolescents are low-dose oral contraceptive pills (OCPs) and non-steroidal anti-inflammatory drugs (NSAIDs) [1, 12]. OCPs suppress ovarian hormone production, inducing decidualization of endometrial tissue, and are known to be safe for long-term use [9]. Gonadotropin-releasing hormone (GnRH) agonists are not recommended for girls younger than 16 years of age due to concerns regarding their adverse effects on bone formation and density [6].

In the present case, we did not immediately begin hormone treatments, since the patient did not experience chronic pelvic pain, and remained asymptomatic after the surgery, as she had yet to reach menarche. However, given that endometriosis is an estrogen-dependent, chronic, progressive disease, the patient may develop symptoms upon the onset of menstruation in the future; therefore, we intend to continue regular follow-ups. Close monitoring, along with collaborative efforts between pediatric surgeons, paediatricians, and gynecologists, is essential to prevent disease recurrence or progression and potential future infertility.

## Conclusion

OE in children, especially before menstruation, is extremely rare, but the possibility of OE should not be excluded from the differential diagnosis, regardless of the patient’s age. The long-term prognosis of pediatric OE diagnosed prior to menstruation, including the possibility of infertility associated with pelvic adhesion, is yet to be determined. It is crucial to maintain regular follow-ups, especially when the patient reaches menarche.

## Data Availability

The data supporting the findings of this study are available from the corresponding author upon reasonable request.

## Abbreviation

OE: Ovarian endometrioma
